# Role of interleukin 4 (IL4) and interleukin 6 (IL6) in the pathogenesis and prognosis of childhood primary immune thrombocytopenia

**DOI:** 10.1007/s00431-023-04945-x

**Published:** 2023-04-25

**Authors:** Marwa Zakaria, Mohamed Beshir, Tamer Hassan, Asmaa Esh, Eman Abdelaziz, Ridha Tayib, Alaa Nafea

**Affiliations:** 1 Pediatrics Department, Faculty of Medicine, Zagazig University, Zagazig, 44519 Egypt; 2 Clinical Pathology Department, Faculty of Medicine, Zagazig University, Zagazig, Egypt

**Keywords:** ITP, IL6, IL4, Cytokines

## Abstract

Immune thrombocytopenia (ITP) is an autoimmune disease characterized by the breakdown of immune tolerance. Impairment of the cellular immunity is primarily evaluated by the levels of the cytokines which can help in predicting the course of ITP. We aimed to assess the levels of IL4 and IL6 in children with ITP and evaluate their role in the pathogenesis and prognosis of this disease. A prospective cohort study was carried on 60 children (15 patients with newly diagnosed ITP, 15 patients with persistent ITP, 15 patients with chronic ITP and 15 healthy children as a control group). Serum IL-4 and serum IL-6 were measured using Human IL-4 and IL-6 ELISA kit in patients and controls. Patients with newly diagnosed and persistent ITP had significantly higher levels of IL4 and IL6 compared to patients with chronic ITP and healthy controls (*p* < 0.001). The mean serum level of IL4 was 762.0, 741.0, 364.6 and 436.8 pg/ml, and the mean serum level of IL6 was 178.5, 164.4, 57.9 and 88.4 pg/ml for patients with newly diagnosed, persistent, chronic ITP and healthy controls respectively. Serum IL-4 was significantly higher in patients who achieved remission than those who did not improve on first line therapy.

*Conclusion*: Serum IL-4 and IL-6 may have a role in the pathogenesis of primary ITP. IL-4 seems to be a good predictor to treatment response.

**Table:** 

**What Is Known:** *• There is a delicate balance of specific cytokine levels in immune thrombocytopenia, which has an important role in the immune system and is known to be deregulated in autoimmune diseases. changes in IL-4 and IL-6 might be involved in the pathogenesis of newly diagnosed ITP in both paediatric and adult patients.* *• We conducted this research study to measure the serum level of IL-4 and IL-6, in newly diagnosed, persistent and chronic ITP patients and study their relation to disease pathogenesis as well as patient’s outcome.* **What Is New:** *• We found that IL4 seems to be a good predictor to treatment response and it was a very interesting observation in our study, and to the best of our knowledge, there is no published data about this finding.*

**What Is Known:**

*• There is a delicate balance of specific cytokine levels in immune thrombocytopenia, which has an important role in the immune system and is known to be deregulated in autoimmune diseases. changes in IL-4 and IL-6 might be involved in the pathogenesis of newly diagnosed ITP in both paediatric and adult patients.*

*• We conducted this research study to measure the serum level of IL-4 and IL-6, in newly diagnosed, persistent and chronic ITP patients and study their relation to disease pathogenesis as well as patient’s outcome.*

**What Is New:**

*• We found that IL4 seems to be a good predictor to treatment response and it was a very interesting observation in our study, and to the best of our knowledge, there is no published data about this finding.*

## Introduction

Primary ITP is defined as a megakaryocytic/platelet-specific autoimmune disorder characterized by isolated thrombocytopenia (platelet count < 100 × 10^9^/L) with or without bleeding manifestations, in the absence of other disorders that may be associated with thrombocytopenia [1]. Mechanisms leading to low platelet count in ITP are multifactorial involving both, increased peripheral platelet destruction and decreased platelet production. Main factors responsible for ITP abnormalities are autoantibodies targeting megakaryocytes and platelet-specific glycoproteins as well as cytotoxic T cells directly acting on platelets [2]. Dysfunction of T cells in ITP may contribute to loss of tolerance, which play a role in the pathogenesis of this disease. T lymphocytes polarize into helper T-1 response characterized primarily by the presence of cytokines interleukin2 (IL2), interferon-γ and tumour necrosis factor (TNF)-α and helper T-2 response produces IL-4, IL-5, IL-6, IL-10 and IL-13. IL-4 is a pleiotropic Th2-type cytokine involved in many immune-modulating functions. IL-4 also drives the development of precursor T-helper cells into the Th2 subset that regulates humoral immunity and Ig production [3]. IL-6 is involved in the production of platelets by activation of megakaryocytes to produce platelets. Furthermore, IL-6 has an inhibitory effect on Treg cell, which could explain the reduction of Treg coinciding with the elevation of IL-6 in ITP patients. Moreover, the elevation of IL-6 coincides with the elevation of Th17 observed previously in ITP patients [4]. To further investigate the role and imbalance of Th cytokines in the pathogenesis and disease outcome, we conducted this research study to measure the serum level of IL-4 and IL-6, in newly diagnosed, persistent and chronic ITP patients and study their relation to disease pathogenesis as well as patient’s outcome.

## Materials and methods

This prospective cohort study was carried on 60 children (their ages ranged from one year to 18 years) with primary ITP who presented to the paediatric haematology unit and outpatient clinic of Zagazig University Hospitals, during the period from March 2020 to September 2020. Patients were divided into to 3 groups: patients with newly diagnosed ITP (*n* = 15), patient with persistent ITP (*n* = 15), patient with chronic ITP (*n* = 15). Fifteen healthy, age- and sex-matched subjects were included as a control group.

### Inclusion criteria

- Approval to sign an informed written consent
- Patients with primary ITP
- Age > 1 year and < 18 years

### Exclusion criteria

- Refusal to sign an informed written consent
- Age < 1 year or > 18 years.
- Patients with secondary immune thrombocytopenia
- Patients with active infection at the time of diagnosis, receiving therapy or drugs causing thrombocytopenia, presence of splenomegaly, any diseases resulting in thrombocytopenia such as SLE

### Diagnosis of ITP

The diagnosis of ITP was made after history taking and physical examination according to international working group (IWG) that defined primary ITP as the presence of isolated thrombocytopenia (platelets count < 100 10^9^/L) with otherwise normal red blood cells and WBCS count in the absence of other disorders that may be accompanied by thrombocytopenia.

### Classifications of ITP

Immune thrombocytopenia was classified as newly diagnosed ITP (within 3 months of diagnosis) persistent ITP (between 3 and 12 months from diagnosis) and chronic ITP (lasting for more than12 months from diagnosis).

Patients were subjected to:

1. Complete physical examination
2. Thorough clinical examination with special emphasis on clinical signs of bleeding
3. Laboratory investigations in the form of complete blood count (CBC) using automated cell counter (Sysmex XN2000, Japan), bone marrow aspiration and examination (when needed)

Patients and controls were subjected to measurement of serum levels of interleukin 4 (IL4) and interleukin 6 (IL6) by ELISA sandwich technique, using commercially available kit (Quantikine, R and D system, Inc., Minneapolis, USA).

### Test principle for IL4, IL6 measurement

The kit uses a double-antibody sandwich enzyme-linked immunosorbent assay (ELISA) to assay the level of both human interleukin 4,6 (IL4,6) in samples. Add interleukin 4,6 (IL4,6) to monoclonal antibody enzyme well which is pre-coated with human interleukin 4,6 (IL4,6) monoclonal antibody, incubation; then, add interleukin 4,6(IL4,6) antibodies labelled with biotin, and combined with streptavidin-HRP to form immune complex; then carry out incubation and washing again to remove the uncombine enzyme. Then, add chromogen solution A, B; the colour of the liquid changes into blue, and at the effect of acid, the colour finally becomes yellow. The chroma of colour and the concentration of the human substance interleukin 4,6 (IL4,6) of sample were positively correlated.

All patients were followed up for 3 months and periodically examined, and their haemoglobin and platelet levels were assessed.

Criteria for assessment of outcome results [5]:

- Complete response (recovery). Platelet count ≥ 100,000/μL persisting for more than 6 weeks after discontinuation of treatment.
- Partial response. Platelet count between 30,000 and 100,000/μL that constitutes an increase relative to baseline persisting for more than 6 weeks after discontinuation of treatment.
- No response (non-recovery). No changes in clinical or biological features.
- Transient response. Initial improvement (clinical or biological) followed by development of new symptoms or a platelet count of less than 30,000/μL in the first 6 weeks after discontinuation of treatment
- Relapse. Platelet count of less than 30,000/μL more than 6 weeks after discontinuation of treatment following a complete or partial response.

### Statistical analysis

Statistical analysis was done using SPSS software version 27 (IBM 2020). Data was presented in tables and figures. Quantitative data was presented as mean, median, standard deviation and range. Qualitative data was presented as frequencies and proportions. The Shapiro–Wilk test was used to determine the distribution characteristics of variables and variance homogeneity. The chi-square test (*χ*^2^) was used to test differences for categorical variables. The Mann–Whitney *U* test (MW) was used to test differences for continuous variables between two groups. The one-way ANOVA test (*F*) was used to test differences when more than two independent groups were present, and variances were equal, while the Kruskal–Wallis test (KW) was used when equal variances were not present. Spearman’s correlation coefficient (*r*) was used to test correlation between IL4, IL6 and continuous variables. The sensitivity, specificity, predictive values, accuracy and likelihood ratios of IL4 as predictor of prognosis were calculated. Receiver operating characteristic (ROC) curves were plotted for the optimal cut-off values of IL4 that was predictive of prognosis as well as the sensitivity and specificity. The optimal cut-off values were defined as the values that allow discrimination between improved and not improved cases with highest sensitivity and specificity. A *p*-value of < 0.05 was accepted as statistically significant (IBM 2020).

### Ethical approval

This study was conducted in accordance with the ethical standards of the Helsinki Declaration of 1964, as revised in 2000. The study protocol number (5828) was approved by the Research Ethics Committee of the Faculty of Medicine, Zagazig University. Informed written consent and/or assent were obtained from the parents or guardians of each child.

## Results

This study included 45 children diagnosed with primary ITP; their age ranges from 1 to 15 years. Patients with chronic ITP patients had significantly higher age and weight compared to patients with newly diagnosed and persistent ITP (*p* < 0.05). The median age was 10, 3 and 7 years respectively. Also, the median weight was 33, 15 and 25 kg respectively. Demographic data is represented in Table 1.

**Table 1:** Demographic data of the studied groups

**Variables**	**Newly diagnosed ITP (*n* = 15)**	**Persistent ITP (*n* = 15)**	**Chronic ITP (*n* = 15)**	**Control group (*n* = 15)**	**Test of sig.**	***p***
Age (years):						
Mean ± SD	5.2 ± 4.4	6.1 ± 3.4	9.6 ± 4.2	6.1 ± 4.2	KW	0.03
Median	3.0	7.0	10.0	5.0	8.6	S
Range	1.0–13.0	2.0–12.0	2.5–15.0	1.5–14.0		
Sex:					χ^2^	
Male	7 (46.7%)	6 (40.0%)	13 (86.7%)	7 (46.7%)	8.3	0.04
Female	8 (53.3%)	9 (60.0%)	2 (13.3%)	8 (53.3%)		S
Weight (kg):					KW	
Mean ± SD	18.4 ± 9.5	22.3 ± 8.6	35.0 ± 18.6	21.2 ± 12.0	9.3	0.02
Median	15.0	25.5	33.0	16.5		S
Range	10.0–37.0	12.5–35.0	12.5–71.0	12.5–46.0		
Consanguinity:					χ^2^	
Positive	3 (20.0%)	3 (20.0%)	5 (33.3%)	4 (26.7%)	1.0	0.8
Negative	12 (80.0%)	12 (80.0%)	10 (66.7%)	11 (73.3%)		

Subcutaneous bleeding was the most common initial clinical presentation in newly diagnosed ITP patients (100%), while it was present in 86.7% of patients with persistent and chronic ITP. The mean initial platelets count was 8.3, 16.9, and 21.4 in patients with newly diagnosed, persistent and chronic ITP respectively with no significant difference (*p* > 0.05). Regarding platelets trend during the follow-up period, newly diagnosed ITP patients had the highest platelet count during follow up period compared to patients with persistent and chronic ITP (247.7 versus 101.4 and 143.0 respectively) with highly significant difference (*p* < 0.05). Clinical and laboratory characteristics are listed in Table 2.

**Table 2:** Clinical history, treatment and outcome in the studied patients

	**Newly diagnosed ** **ITP (*n* = 15)**	**Persistent ITP ** **(*n* = 15)**	**Chronic ITP ** **(*n* = 15)**	**Test ** **of sig.**	***p***
Age at diagnosis (years):				KW	
Mean ± SD				0.3	0.8
Median	5.2 ± 4.4	5.4 ± 3.4	4.6 ± 2.7		
Range	3.0	6.0	5.0		
	1.0–13.0	1.5–11.0	1.0–9.0		
Subcutaneous bleeding:				χ^2^	
Yes	15 (100%)	13 (86.7%)	13 (86.7%)	2.2	0.3
No	0 (0.0%)	2 (13.3%)	2 (13.3%)		
Bleeding per orifice:				χ^2^	
Yes	8 (53.3%)	12 (80.0%)	11 (73.3%)	2.7	0.3
No	7 (46.7%)	3 (20.0%)	4 (26.7%)		
Initial platelet count:				KW	
Mean ± SD	8.3 ± 5.7	16.9 ± 17.3	21.4 ± 15.6	4.4	0.1
Median	9.0	6.0	18.0		
Range	1.0–18.0	2.0–51.0	1.0–48.0		
Initial blood haemoglobin:				F	
Mean ± SD	10.4 ± 1.1	10.9 ± 1.9	10.9 ± 1.6	0.6	0.6
Follow up platelet count:				KW	
Mean ± SD	247.7 ± 141.6	101.4 ± 87.4	143.0 ± 116.1	9.1	0.01
Median	320.0	65.0	130.0		S
Range	8.0–420	8.0–260.0	17.0–300		
Treatment					
Observations	2 (13.3%)	0 (0.0%)	0 (0.0%)	4.2	0.1
Corticosteroid	10 (66.7%)	13 (86.7%)	10 (66.7%)	2.0	0.3
IVIG	10 (66.7%)	15 (100%)	8 (53.3%)	8.9	0.01(S)
TPO oral	0 (0.0%)	5 (33.3%)	10 (66.7%)	11.3	0.002(S)
TPO SC	0 (0.0%)	2 (13.3%)	4 (26.7%)	4.6	0.09
Rituximab	0 (0.0%)	0 (0.0%)	1 (6.7%)	2.0	0.3

Of patients with newly diagnosed ITP and chronic ITP, 66.7% received corticosteroids as the first-line therapy versus 86.7% in patients with persistent ITP with no significant difference (*p* > 0.05). All patients with persistent ITP had received IVIG compared to 66.7% and 53.3% in newly diagnosed and chronic ITP patients. Almost two-thirds of patients with chronic ITP had received oral TPO compared to 33.3% and 0% in persistent and newly diagnosed ITP with highly significant difference (*p* < 0.05). Different treatment lines are listed in Table 2.

Regarding disease outcome, 93.3% of newly diagnosed patients achieved complete remission after first-line therapy. Only one patient did not achieve complete remission.

Regarding the levels of IL4 and IL6 in our studied groups, IL4 was significantly higher in patients with newly diagnosed and persistent ITP compared to patients with chronic ITP and healthy controls. The mean serum level of IL4 was 762.0, 741.0, 364.6 and 436.8 pg/ml for patients with newly diagnosed, persistent, chronic ITP and healthy controls respectively. Also, IL6 was significantly higher in patients with newly diagnosed and persistent ITP compared to patients with chronic ITP and healthy controls. The mean serum level of IL6 was 178.5, 164.4, 57.9 and 88.4 pg/ml for patients with newly diagnosed, persistent, chronic ITP and healthy controls respectively. Results are represented in Table 3.

**Table 3:** IL4 and IL6 in the studied patients

**Variables**	**Newly diagnosed ITP (*n* = 15)**	**Persistent ITP (*n* = 15)**	**Chronic ITP (*n* = 15)**	**Control group (*n* = 15)**	**KW**	***p***
IL4:						
Mean ± SD	762.0 ± 307.7	741.0 ± 205.7	364.6 ± 199.6	436.8 ± 292.1	21.4	< 0.001
Median	757.4*	680.3*	373.1	357.2		HS
Range	139.3–1187.2	480.3–1142.6	138.6–751.1	235.0–889.4		
IL6:						
Mean ± SD	178.5 ± 119.3	164.4 ± 97.1	57.9 ± 31.5	88.4 ± 57.1	16.9	< 0.001
Median	127.4*	115.7*	58.9	62.4		HS
Range	20.4–324.3	80.2–333.1	25.9–96.7	34.8–178.9		

IL4 was significantly higher in patients who achieved remission after first-line therapy compared to those who did not improve on first-line therapy (696.0 versus 459.6 pg/ml, *p* = 0.01). No significant relationship was found between IL4 levels and any of patient’s characteristics, bleeding profile or type of treatment received. Similarity, we did not find significant relationship between IL6 levels and patient’s characteristics or bleeding profile (Table 4).

**Table 4:** Association between IL4, IL6 and characteristics, clinical presentation, treatment and outcome of newly diagnosed ITP patients

**Variables**	**IL4**					**IL6**				
	Mean ± SD	Median	Range	**MW**	**P**	Mean ± SD	Median	Range	**MW**	**P**
Sex:										
Male	573.1 ± 292.1	555.3	168.9 – 1142.6	1.3	0.2	129.0 ± 104.5	96.2	20.4 – 333.1	0.3	0.8
Female	690.1 ± 306.6	697.9	138.6 – 1187.2			140.0 ± 105.7	90.4	24.8 – 309.5		
Consanguinity:										
Positive	598.0 ± 168.4	706.6	371.5 – 757.4	0.1	0.9	85.0 ± 31.9	88.8	28.0 – 139.6	1.3	0.2
Negative	630.4 ± 334.1	668.9	138.6 – 1187.2			149.4 ± 114.3	96.2	20.4 – 333.1		
subcutaneous bleeding:										
Yes	630.2 ± 309.9	680.3	138.6 – 1187.2	0.5	0.7	138.1 ± 107.6	95.5	20.4 – 333.1	0.6	0.6
No	543.6 ± 188.0	543.6	380.7 – 706.4			87.3 ± 32.8	87.3	58.9 – 115.7		
Bleeding per orifice:										
Yes	591.2 ± 311.6	591.5	139.3 – 1047.9	0.1	0.9	128.1 ± 100.3	95.6	20.4 – 333.1	1.4	0.2
No	691.9 ± 272.5	754.3	138.6 – 1031.9			145.8 ± 114.7	93.0	25.9 – 324.3		
Corticosteroid:										
Yes	633.0 ± 269.8	680.3	138.6 – 1187.2	0.1	0.9	132.4 ± 92.0	95.6	20.4 – 324.3	1.2	0.2
No	593.6 ± 385.2	569.1	139.3 – 1142.6			136.9 ± 136.5	64.1	24.8 – 333.1		
IVIG:										
Yes	665.1 ± 309.4	680.3	138.6 – 1187.2	1.2	0.2	156.2 ± 111.3	115.7	24.8 – 33.1	2.4	0.01
No	505.3 ± 250.2	536.9	168.9 – 757.4			71.5 ± 38.2	78.3	20.4 – 125.9		S
TPO oral:										
Yes	611.6 ± 375.4	480.3	179.3 – 1187.2	0.3	0.8	150.7 ± 126.0	88.8	26.9 – 333.1	0.1	0.9
No	629.1 ± 252.2	684.9	138.6 – 1031.9			123.3 ± 89.0	96.2	20.4 – 324.3	0	
TPO SC:										
Yes	409.8 ± 154.7	384.3	138.6 – 708.4	1.7	0.09	76.3 ± 41.0	87.2	25.9 – 115.7	1.5	0.1
No	655.2 ± 296.4	680.3	139.3 – 1187.2			142.4 ± 108.2	95.5	20.4 – 333.1		
Improvement	696.0 ± 305.0	697.7	139.3 – 1187.2	2.3	0.01S	145.4 ± 109.0	95.5	20.4 – 333.1	1.0	0.3
No improvement	459.6 ± 222.9	432.3	138.6 – 906.6			107.7 ± 90.2	88.0	26.9 – 310.1		

IL6 was higher in patients who received IVIG. The mean IL6 was 156.2 pg/ml in patients who received IVIG compared to patients who received corticosteroids or thrombopoietin agonist (132.4 and 150.7 pg/ml, *p* = 0.01). IL6 was higher in patients who achieved remission after first-line therapy compared to those who did not improve on first-line therapy (145.4 versus 107.7 pg/ml), but the difference did not reach statistically significant level (Table 4).

**Fig. 1:**
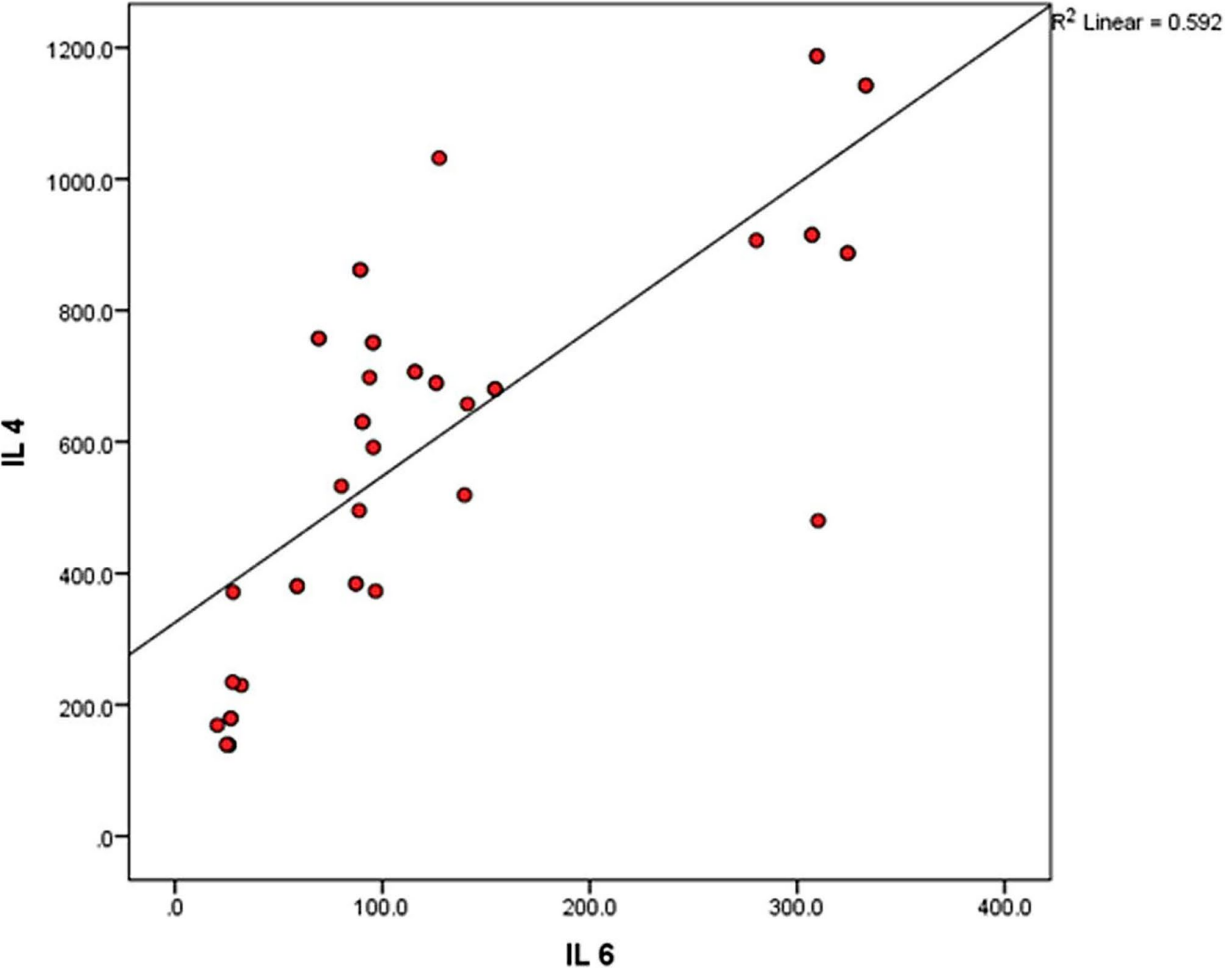
Shows the correlation between IL4, IL6 and continuous variables of the studied ITP patients

There was significant positive correlation between levels of IL4 and IL6 (*r* = 0.59, *p* = 0.001) (Fig. 1). There was negative correlation between IL4 and platelet count at initial diagnosis, but this correlation did not reach a statistically significant level (*r* =  − 0.21). The same was observed with IL6 and initial platelet count (*r* =  − 0.13).

**Fig. 2:**
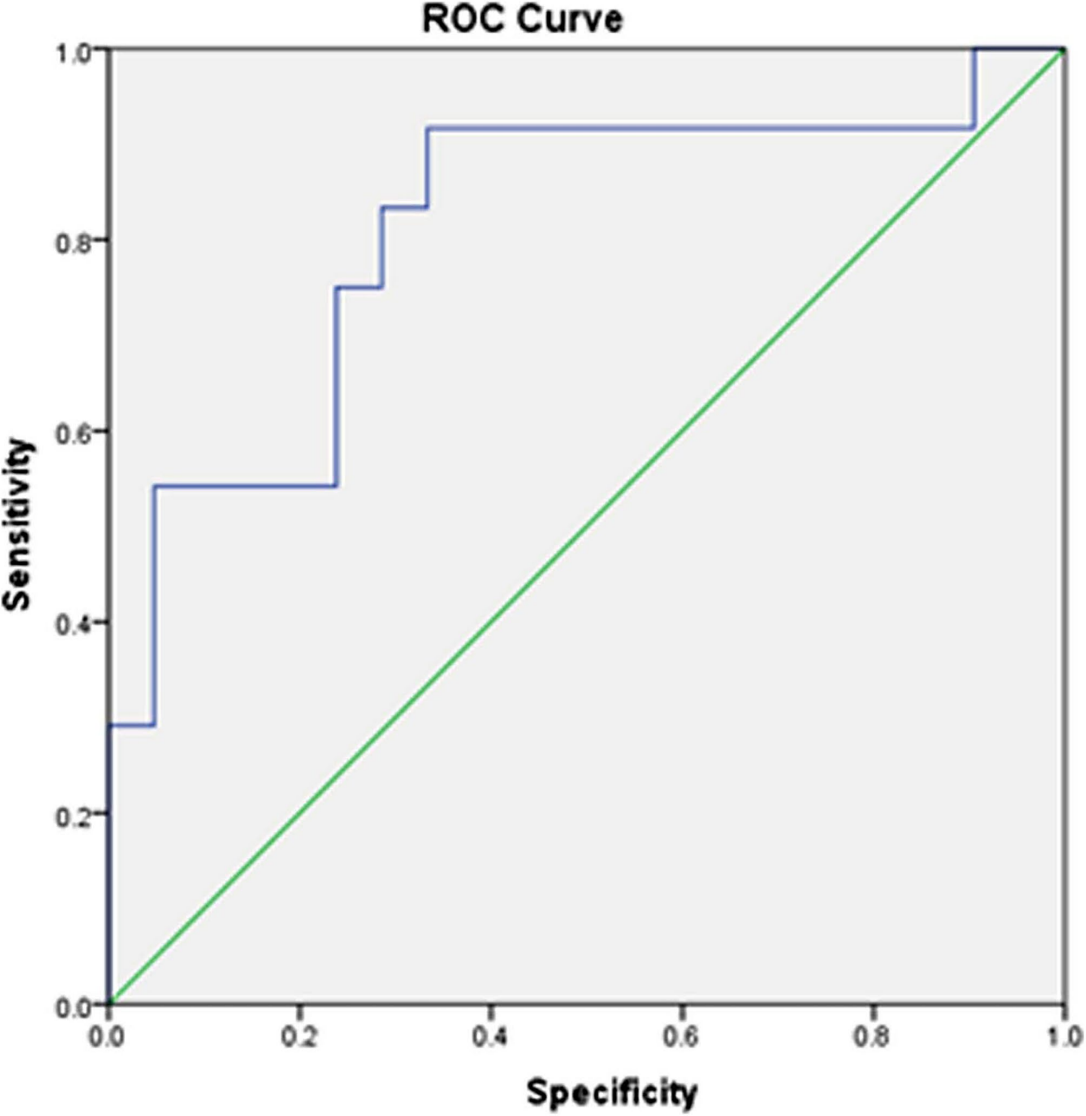
Shows the prognostic performance of IL4

At cut off point of 507.4, IL4 had 91.7% sensitivity, 66.7% specificity and 80.0% accuracy in predicting prognosis. Positive and negative predictive values were 75.9% and 87.5% respectively. Likelihood ratios positive and negative were 2.8 and 0.12 respectively, indicating a fair prognostic performance. Area under the curve was statistically significant (Fig. 2).

## Discussion

Dysfunction of T-cells may contribute to loss of tolerance in patients with ITP, and impairment of the delicate balance of specific cytokines may play a role in the pathogenesis of this disease. T lymphocytes polarize into Th-1 response characterized primarily by the presence of cytokines IL2, interferon-γ and tumour necrosis factor (TNF)-α, and Th-2 response produces IL-4, IL-5, IL-6, IL-10, and IL-13 [6].

Understanding the role of T-cell subsets will permit a better control of autoimmunity through manipulation of their cytokine network. Dysfunction at cellular immunities is evaluated by the levels of cytokine. Measurement of levels of cytokines may help in prediction of the course of ITP [7].

The present study showed that there was a significant difference between the studied groups in age and sex. In our study, patients with chronic ITP were older than patients with newly diagnosed and persistent ITP, with statistically significant difference *p* > 0.05. This agreed with the study of Ebeid et al., where patients with chronic ITP were older, with mean age of 7.7 ± 4.19, than those with acute ITP, with mean age of 3.95 ± 2.21(χ^2^ = 3.83, *p* = 0.001), and this is most probably because of the chronic nature of the disease. As children with the chronic form of the disease are diagnosed for a much longer period, children with acute ITP are usually diagnosed at a younger age [6].

Regarding platelet trend during the follow-up period, newly diagnosed ITP patients had the highest platelets count during follow-up period compared to patients with persistent and chronic ITP, 247.7 ± 141.6 versus 101.4 ± 87.4 and 143.0 ± 116.1 respectively with highly statistically significant difference *p* < 0.05.

Similarly, Gözmen et al. found that post treatment platelet count was higher than the pre-treatment platelet counts in newly diagnosed ITP group (*p* < 0.00) [8]. Contrarily, Ebeid et al. reported that there was no statistically significant difference in platelet count during follow up period [6].

In the present study, we found a significant difference between the studied ITP patients regarding the received treatment, where all patients with persistent ITP received IVIG compared to 66.7% and 53.3% in newly diagnosed and chronic ITP patients respectively. Almost two-thirds of patients with chronic ITP received oral TPO compared to 33.3% and 0% in persistent and newly diagnosed ITP with highly significant difference (*p* < 0.05) while 66.7% of patients with newly diagnosed ITP and chronic ITP received corticosteroids as the first-line therapy versus 86.7% in patients with persistent ITP with no significant difference(*p* > 0.05). This was consistent with those reported by Ebeid et al. [6].

Regarding disease outcome, 93.3% of newly diagnosed patients achieved complete remission after first-line therapy. Only one patient did not achieve complete remission. This is consistent with data reported by Yacobovich et al. who reported that childhood immune thrombocytopenia is usually a self-limited disorder lasting for a few weeks or months, but in approximately 25–30% of the children, the condition becomes chronic [9].

In our study, IL4 was significantly higher in patients with newly diagnosed and persistent ITP compared to patients with chronic ITP and healthy controls.

Our results were matched with Talaat et al. who found that IL-4 was significantly higher in ITP patients (with maximum elevation in the acute stage of the disease) compared to controls and found a positive correlation between the acute form of ITP and IL-4 (*r* = 0·487, *p* < 0·05) [4]. Also, our results are in agreement with Webber et al. who reported that IL-4 plays an important role in autoantibody production [10]. Additionally, Culić et al. in their study on adult ITP patient found significantly higher levels of IL4 in patients compared to controls [11].

On the contrary, Jernas et al. found that IL4 levels were higher in patients with chronic ITP than those with newly diagnosed ITP [12]. Wu et al. were in contrast with our results and found that serum level of IL-4 in patients with ITP before treatment was significantly lower than those in healthy subjects (*p* < 0.05) [13].

Higher levels of IL4 in newly diagnosed ITP patients that were observed in our study as well as a considerable number of studies could be explained based on the fact that this cytokine is produced by Th2 lymphocytes which are involved in autoantibody production. IL-4 is essential for T-dependent B-cell differentiation and isotype switching to several IgG isotypes including antiplatelet antibodies [14].

Also, higher levels of IL4 could be related to activation of macrophages, which have been reported to be stimulated in ITP patients by platelet autoantigen and lead to activation of T cells. MoreIL-4 secretion was induced by activated T cells, which may lead to the activation of B cells, a remarkable feature of ITP [8].

Also, the increase in Th2 cytokine (IL-4 and IL-10) levels can affect differentiation and survival of pathogenic B cells in ITP patients [10].

The change in the nomenclature of the different phases of ITP might explain the divergent results between us and Jernas et al. where chronic ITP was formerly defined as persistence of thrombocytopenia beyond 6 months of diagnosis, but later, it was defined as persistence of thrombocytopenia beyond 12 months of diagnosis.

IL4 was significantly higher in patients who achieved remission after first-line therapy compared to those who did not improve on first-line therapy. IL4 had a fair prognostic performance where at cut-off point of 507.4 pg/ml, sensitivity was 91.7%, specificity was 66.7%, and accuracy was 80.0%. Also, positive and negative predictive values were 75.9% and 87.5% respectively. No significant relationship was found between IL4 levels and any of patient’s characteristics, bleeding profile or type of treatment received. Similarity, we did not find significant relationship between IL6 levels and patient’s characteristics or bleeding profile.

This was a very interesting observation in our study, and to the best of our knowledge, there is no published data about this finding. Further studies are needed to support this prognostic significance of IL4.

Also, IL6 was significantly higher in patients with newly diagnosed and persistent ITP compared to patients with chronic ITP and healthy controls.

Our results were matched with a previous Egyptian study conducted by Ebeid et al. who found significantly higher IL6 levels in patients with acute ITP than those with chronic ITP [6]. Similarly, Yalinbas et al. found that newly diagnosed ITP patients had significantly higher T helper intracellular IL-2, IL-4, IL-6 and IFN-γ percentages compared with the control group [15].

On the contrary, Del Vecchio et al. found no significant difference between patients with newly diagnosed and chronic ITP as regards IL6 levels [14].

IL-6 has an inhibitory effect on Treg cells, which could explain the reduction of Tregs coinciding with the elevation of IL-6 in ITP patients. Moreover, the elevation of IL-6 coincides with the elevation of Th17 observed previously in ITP patients [16]. Previous study speculated that elevated IL-6 may be related to compensatory megakaryopoiesis and thrombopoiesis, and it is decreased with response to treatment [17]. This was observed in our study where patients who achieved remission after first therapy had lower levels of IL-6 though not reached to a statistically significant level.

In our study, there was significant positive correlation between levels of IL4 and IL6 (*r* = 0.59, *p* = 0.001). There was negative correlation between IL4 and platelet count at initial diagnosis, but this correlation did not reach a statistically significant level (*r* =  − 0.21). The same was observed with IL6 and initial platelet count (*r* =  − 0.13).

Positive correlation between IL4 and IL6 is a logic observation as both cytokines were significantly elevated in newly diagnosed patients compared to chronic patients. Ebeid et al. found a statistically significant negative correlation between IL-6 and platelets count (*r* =  − 0.7 and *p* < 0.001) [6].

In our study, there was significant association between prognosis and patient’s characteristics where young age, weight and elevated platelet count during follow up were associated with good prognosis and improved patients’ outcome, while there was no significant association between sex and prognosis and this can be explained by the fact that the newly diagnosed patients had better prognosis than chronic cases as newly diagnosed cases were younger in age and had less weight.

## Conclusion

Serum IL4 and IL6 may have a role in the pathogenesis of primary ITP. IL4 seems to be a good predictor to treatment response. Wide-scale studies should be performed to validate the prognostic significance of IL4 so that it can be added to already existing prediction scores to improve their clinical prediction.

Further studies are still needed to clarify the prognostic significance of IL6.

## Data Availability

Data are avaliable upon reasonable request from the corsponding author.

## Abbreviations

IL: Interleukin
ITP: Autoimmune thrombocytopenia
Th17: T helper 17
IVIG: Intravenous immune globulins
TNF: Tumour necrosis factor
Th: T-helper
Treg: T regulatory
SLE: Systemic lupus erythematosus
